# ZDHHC1 downregulates LIPG and inhibits colorectal cancer growth via IGF2BP1 Palmitoylation

**DOI:** 10.1038/s41417-024-00808-1

**Published:** 2024-07-28

**Authors:** Qun Zhang, Zhouyuan Du, Wei Zhou, Wei Li, Qinglin Yang, Haixin Yu, Tao Liu

**Affiliations:** Department of Digestive Surgical Oncology, Union Hospital, Tongji Medical College, Huazhong University of Science and Technology, Wuhan, 430022 China

**Keywords:** Cancer, Cell biology

## Abstract

Alteration in lipid metabolism is recognized as a hallmark feature of colorectal cancer (CRC). Protein S-palmitoylation plays a critical role in many different cellular processes including protein-lipid interaction. Zinc Finger DHHC-Type Containing 1 (ZDHHC1, also known as ZNF377) belongs to the palmitoyl-transferase ZDHHC family, and is a potential tumor suppressor. However, our knowledge of the functional roles of ZDHHC1 in CRC is limited. We discovered that ZDHHC1 expression was downregulated in CRC tissues and that low levels of ZDHHC1 were associated with unfavorable prognosis. Functional studies showed that ZDHHC1 inhibited CRC cell proliferation and invasion in vitro and in vivo. We also found that lipase G (LIPG) is negatively regulated by ZDHHC1 and plays a key role in CRC cell growth through lipid storage. Additionally, we demonstrated that ZDHHC1 functions as a IGF2BP1-palmitoylating enzyme that induces S-palmitoylation at IGF2BP1-C337, which results in downregulated LIPG expression via m6A modification. Mechanistic investigations revealed that the ZDHHC1/IGF2BP1/LIPG signaling axis is associated with inhibition of CRC cell growth. Our study uncovers the potential role of ZDHHC1 in CRC, including inhibition of CRC growth by reducing the stability of LIPG mRNA in an m6A dependent-manner by palmitoylation of IGF2BP1.

## Introduction

Colorectal cancer (CRC) ranks as the third most commonly diagnosed cancer and the third most common cause of cancer‐related death. Surgical resection is a primary therapeutic approach in the treatment of CRC [1, 2]; however, stage at diagnosis dictates whether surgery is a feasible option. In addition, stage at diagnosis is the most important predictor of survival, with 5 year relative survival ranging from 91% for localized disease to 14% for patients with distant disease [1, 3, 4]. Lipids are essential components of the cell membrane which play a role in regulating cell survival, proliferation, and a range of other cellular processes [5]. Lipids are also important for metabolism [6, 7]. Alterations in lipid metabolism are recognized as a hallmark feature of CRC [8–10]. Therefore, clarifying the molecular mechanisms that underlie lipid uptake in CRC cells is likely to be of great importance for the development of targeted therapeutic strategies and improvement in patient prognosis.

Protein S-palmitoylation is a reversible post-translational modification (PTM) whereby palmitic acid, a 16-carbon long saturated fatty acid, is covalently attached to proteins at cysteine residues via a labile thioester bond [11, 12]. Protein S-palmitoylation is known to play critical roles in many different cellular processes including protein-lipid interaction, which is catalyzed by polytopic transmembrane proteins named protein acyltransferases (PATs) with zinc-finger and aspartate–histidine–histidine–cysteine (DHHC) motifs [13–15]. There are 23 distinct members of the ZDHHC family in humans, and different ZDHHC enzymes may act as either oncoproteins or tumor suppressors depending on the specific substrate [16, 17]. Zinc Finger DHHC-Type Containing 1 (ZDHHC1, also known as ZNF377) belongs to the palmitoyl-transferase ZDHHC family, and is a potential tumor suppressor protein [18]. Evidence has shown that ZDHHC1 localizes on the endoplasmic reticulum (ER) and membranous structures in the cell [5]. However, there are limited reports on S-palmitoylation mediated by ZDHHC1 in lipid uptake in CRC cells.

In this study, we identified a direct correlation between decreased expression of ZDHHC1 and decreased survival in patients with CRC. We also found that ZDHHC1 suppresses CRC cell growth. Next, we revealed that lipase G (LIPG) is negatively regulated by ZDHHC1 and plays a key role in CRC cell growth through lipid storage. Mechanistically, we identified LIPG as a direct target of IGF2BP1, and showed that ZDHHC1 is a IGF2BP1-palmitoylating enzyme that induces S-palmitoylation at IGF2BP1-C337, which leads to downregulated expression of LIPG via m6A modification. These findings suggest a novel mechanism underlying the development of CRC and provides potential therapeutic targets for the treatment of patients with CRC.

## Methods

### Data mining and analysis tools

The levels of twenty-three ZDHHC enzymes were assessed using data from The Cancer Genome Atlas (TCGA) and the Genotype-Tissue Expression (GTEx) dataset. The 3-year overall survival in CRC patients was examined using TIMER 2.0 (http://timer.cistrome.org). To predict m6A modification sites on the RNA sequences, SRAMP (http://www.cuilab.cn/sramp/), a motif-dependent predictor, was employed.

### Cell lines and cell culture

Colorectal cancer cells (1 × 10^4^) were seeded in 96-well plates with MTS solution added for cell growth assessment (cat. no. ab197010; Abcam). Absorbance at 490 nm was measured. For colony formation, cells (500 cells/well) were seeded in 6-well plates with 10% fetal bovine serum (FBS) complete medium, then fixed in methanol, stained with 1% Crystal Violet for 30 min, and colony count determined after washing with PBS.

### Cell transfection and plasmid construction

Transfections were carried out with Lipofectamine 2000 (Thermo Fisher Scientific, Waltham, MA, USA), using shRNAs (Sigma-Aldrich, Shanghai, China) to generate various lentiviral particles in 293 T cells. After 24 h, the cell culture medium was refreshed. The virus-containing medium was then harvested 48 h later and utilized for CRC cell transduction following the addition of 12 ug/mL polybrene. Puromycin selection (10 ug/mL; 24 h) was subsequently applied to remove non-infected cells. The sequences of the shRNAs were as follows: ZDHHC1-shRNA#1, 5′-CCGGGCACAAGCTCACCACCTATGACTCGAGTCATAGGTGGTGAGCTTGTGCTTTTTG -3′;

ZDHHC1-shRNA#2, 5′-CCGGGCTCTGCTTCCACATTTATCTCTCGAGAGATAAATGTGGAAGCAGAGCTTTTTG-3′;

IGF2BP1-shRNA, 5′-CCGCCUUAAAGGAUGGUUCAUUUCGAAAAAUGAACCAUCCUUUAAGGC-3′; LIPG-shRNA#1,

5′-GCCTTTCAGAGTTTACCAT-3′;

LIPG-shRNA#2, 5′-GCCGCAAGAACCGTTGTAA-3′.

Flag-ZDHHC1 and Flag-LIPG plasmids were cloned into the CMV-MCS-3xFlag-SV40-neomycin vector by GENECHEM (Shanghai, China). All plasmids were verified by sequencing.

### Quantitative real-time polymerase chain reaction (qRT-PCR)

Colorectal cancer cell total RNA was extracted utilizing TRIzol (Thermo Fisher Scientific), following the manufacturer’s instructions, and reverse transcribed into cDNA using Primescript RT Reagent (Takara, Japan). Real-time PCR was conducted using a 7500 Real-time PCR System (Applied Biosystems) with the SYBR Premix Ex Taq Kit (Takara). The following primers were used: Gapdh, forward: 5′-CAGCGACACCCACTCCTC-3′, reverse: 5′-TGAGGTCCACCACCCTGT-3′;

ZDHHC1, forward: 5′-GCCCTGCTCATCCTTCTG-3′, reverse: 5′-CGCATCTTGGGAGGACAT-3′;

LIPG: forward: 5′-AATCAGGACAAGCCGAGT-3′, reverse: 5′-GCCAATGCTATTACAACG-3′;

IGF2BP1: forward: 5′- GCGGCCAGTTCTTGGTCAA-3′, reverse: 5′-TTGGGCACCGAATGTTCAATC-3′.

### In vitro cell growth assay

Colorectal cancer cells (1 × 10^4^) were seeded in 96-well plates with MTS solution (cat. no. ab197010; Abcam) added for growth assessment via absorbance at 490 nm. For colony formation, cells were seeded in 6-well plates (500 cells/well) and cultured in complete medium with 10% FBS at 37 °C. After 2 weeks, cells were fixed in methanol for 30 min, stained with 1% Crystal Violet for 30 min, washed thrice with PBS, and colonies were counted.

### Invasion assay

The in vitro cell invasion assays utilized Bio-Coat Matrigel invasion chambers (BD Biosciences, Beijing, China) following the provided guidelines. Cells were allowed to grow in the chamber inserts for 24 h, then fixed in methanol for 15 min and stained with crystal violet for 20 min. The number of invading cells was quantified by counting at least three fields per group.

### Tumorigenicity in vivo

Approval for all animal procedures was granted by the Ethics Committee of Tongji Medical College, Huazhong University of Science and Technology ([2022] Approval IACUC Number: 3823). Cg-Foxn1nu/Crl mice, specifically bred for BALB/c nude strains, were sourced from Vitalriver (Beijing, China). HCT116 cells were subcutaneously injected into 4 week-old male nude mice which was randomly assigned to different group at a concentration of 2 × 10^6^ cells in 100 μl of PBS. Tumor dimensions were measured bi-dimensionally using vernier calipers every 2 days until euthanasia of the mice, which occurred after 3 weeks. The volume of the implanted tumor was calculated using the formula: tumor volume = length × width^2^ × 0.5.

### Tissue microarray and immunohistochemistry (IHC)

Tissue microarray (cat. no. D026Co01; Zhongke Guanghua Intelligent Biotechnology Co., LTD, Xian, China) and immunohistochemistry (IHC) were conducted to evaluate ZDHHC1 and LIPG levels in colonic adenocarcinoma. Antibodies used were ZDHHC1 (26545-1-AP; Proteintech; 1:300 dilution) and LIPG (67434-1-lg; Proteintech; 1:300 dilution). IHC scores, based on staining intensity and proportion of positive tumor cells, were determined independently by two blinded experienced pathologists.

### Co-immunoprecipitation and immunoblotting

For co-immunoprecipitation, cells were harvested and incubated in 1 mL of RIPA buffer on ice for 20 min. After centrifugation at 12,000 g for 15 min at 4 °C, the supernatant was collected and mixed with Pierce Protein G Agarose (Thermo Fisher Scientific) along with primary antibody or IgG control overnight at 4 °C. Beads were washed five times with RIPA buffer, then resuspended with loading buffer and boiled at 100 °C for 5 min. The supernatant was subjected to immunoblotting. For immunoblotting, cell extracts were collected in lysis buffer and proteins were separated by SDS–polyacrylamide gel electrophoresis before transferring to PVDF membranes. After blocking with PBS containing 5% BSA, the membrane was incubated with primary antibody overnight at 4 °C, followed by incubation with HRP-conjugated anti-mouse or anti-rabbit IgG for 2 h at room temperature. Protein bands were detected using an enhanced chemiluminescence (ECL) detection system per the manufacturer’s instructions. Primary antibodies used included anti-ZDHHC1, anti-LIPG, and anti-IGF2BP1 (Proteintech).

### RNA binding protein immunoprecipitation (RIP)

Cells were lysed with IP lysis buffer (P0013J, Beyotime, Shanghai, China) supplemented with protease inhibitor and RNase inhibitor on ice for 30 min. After centrifugation at 12,000 g for 10 min, lysates were divided into two parts: one for whole cell extraction (input group) and the other for immunoprecipitation (IP). For the IP group, lysates were incubated with 5 μg anti-IGF2BP1 (22803-1-AP, Proteintech, China) or IgG antibody (14678–1-AP, Proteintech, China) overnight at 4 °C. Protein A/G magnetic beads (Bimake, China) were washed and mixed with lysate-antibody complexes, rotated at 4 °C for 6 h. RNA-protein complexes were washed with elution buffer and treated with proteinase K at 55 °C for 1 h. Bound RNAs were extracted and analyzed by RT-qPCR for quantitative assessment, with relative enrichment normalized to the input.

### MeRIP-qPCR

Total RNA was extracted as previously described, with 10% reserved for the input control and the remaining RNA used for m6A-IP. Antim6A antibody (ab151230, abcam, USA) or mouse IgG was coupled to magnetic beads using the Dynabeads™ Antibody Coupling Kit (14311D, Invitrogen, USA) per the manufacturer’s instructions. Subsequently, RNA was incubated with antibody-conjugated beads in 500 μl binding buffer at 4 °C for 4 h with continuous rotation. M6A-modified mRNAs were eluted from the beads with elution buffer for further purification and analysis by RT-qPCR. Relative enrichment was normalized to the input.

### Luciferase reporter assay

Cells were initially seeded into 24-well plates and cultured for 24 h. Subsequently, plasmids carrying either wild-type or mutated-type LIPG were transfected into the cells. Following transfection for 12 h, cells were re-seeded into 96-well plates and further incubated for 24 h. The Dual-Luciferase® Reporter Assay System (E1910, Promega, USA) was employed to analyze luciferase activity, with Renilla Luciferase (R-luc) utilized for the normalization of firefly luciferase (F-luc) activity.

### mRNA stability assay

Cells were seeded in 6-well plates and allowed to reach ~50% confluence after 24 h of incubation. Subsequently, the cells were treated with actinomycin D (5 μg/ml, Sigma, USA) and harvested at 0, 6, and 12 h. Total RNA was extracted and subjected to qRT-PCR analysis. The mRNA levels were quantified and normalized to GAPDH expression at each time point.

### ABE assay

ABE assay was performed according to the manufacturer’s instructions (AM10314, AIMSMASS).

### Triacylglyceride quantification and visualization of lipid droplets

Cellular triglyceride levels were assessed utilizing the Triglyceride Quantification Kit (Abcam) in accordance with the manufacturer’s protocol. Fluorescence emission (Ex/Em 535/587 nm) was measured using a Tecan Infinite 200PRO plate reader (Tecan Group AG). For visualizing lipid droplets, cells were stained with BODIPY 493/503 (C2053S, Beyotime) for 20 min following fixation and permeabilization.

### Statistical analysis

Statistical analyses were performed using GraphPad Prism (version 7.0, GraphPad software, USA) and SPSS 21.0 software (IBM, SPSS Statistics, USA). The two-tailed Student’s *t*-test was employed to compare results between two groups, while one-way ANOVA was utilized for multiple comparisons. All data are expressed as mean ± standard deviation (SD) from three independently conducted experiments. Statistical significance was determined as *P* < 0.05 (*P < 0.05, **P < 0.01, ***P < 0.001, ****P < 0.0001, ns: not statistically significant).

## Results

### ZDHHC1 low expression is correlated with poor prognosis in CRC

**Fig. 1:**
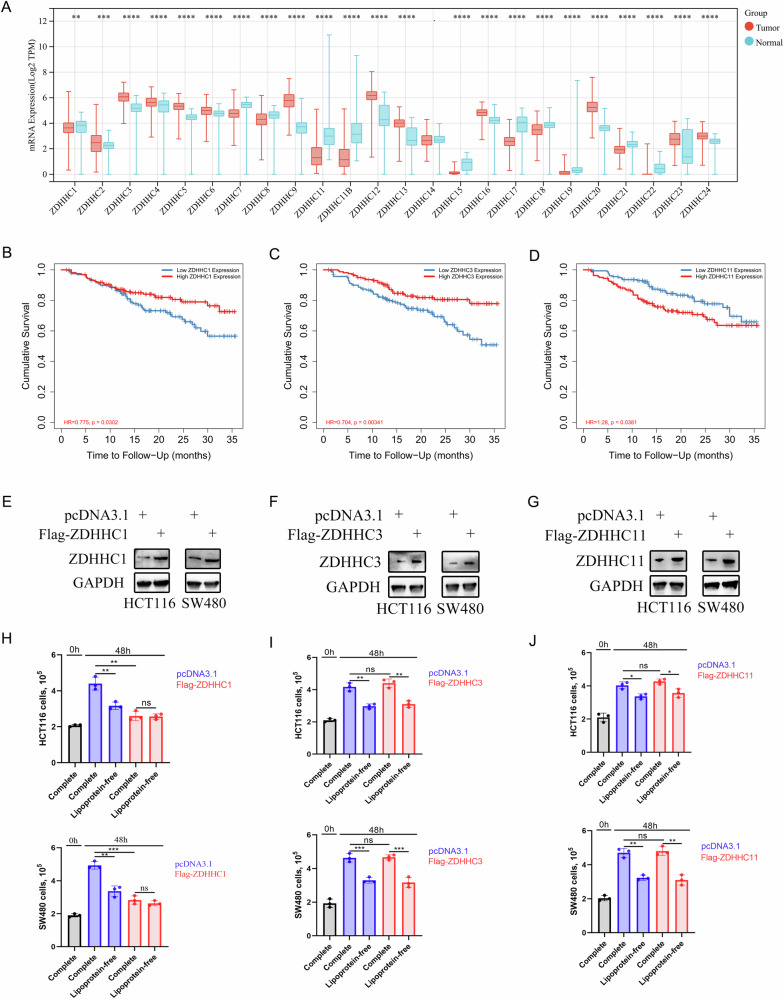
ZDHHC1 low expression is correlated with poor prognosis in CRC. The expression of ZDHHC family members in CRC were presented by box-plot analyses (**A**). Three-years overall CRC survival of ZDHHC1, ZDHHC3 and ZDHHC11 were analyzed via TIMER 2.0 (**B**–**D**). Cells were harvested for WB analysis (**E**–**G**). HCT116 and SW480 cell growth for 48 h in complete medium: medium containing 10% FBS; lipoprotein-free medium: medium containing 10% free lipoprotein FBS (**H**–**J**). Data presented as mean ± SD with three replicates. Student’s *t*-test was used to determine the statistical significance. *p < 0.05; **p < 0.01; ***p < 0.001.

To investigate the relationship between the expression of ZDHHC family members and CRC, we analyzed ZDHCCs’ expression level in CRC patients through TCGA dataset and GTEx dataset. The data distributions were presented by box-plot analyses. We found that only ZDHHC14 mRNA expression in CRC tissues has not changed compared with levels found in nomal tissues (Fig. 1A). Then, we analyzed 3-year overall survival of CRC patients via TIMER 2.0 (Fig. S1) and therefore screened for three genes (ZDHHC1, ZDHHC3 and ZDHHC11) that were statistically significant (Fig. 1B–D). These three genes were all overexpressed in CRC cells and then verified by western blot (WB) (Fig. 1E–G). Finally, we found that overexpression of ZDHHC1 could affect CRC cell proliferation. We also confirmed that the growth of HCT116 and SW480 cells is impaired in lipoprotein-depleted media (Fig. 1H–J).

### ZDHHC1 inhibits CRC cell proliferation and invasion

**Fig. 2:**
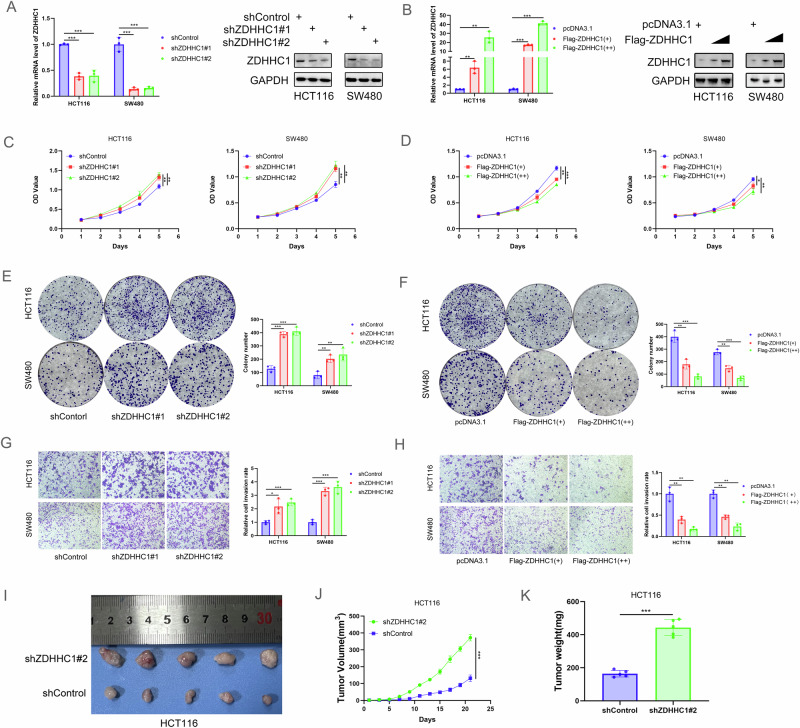
ZDHHC1 inhibits CRC cell proliferation and invasion. HCT116 and SW480 cells were infected with indicated shRNAs. Cells were harvested for WB and RT-qPCR analysis (**A**), MTS assay (**C**), colony formation assay (**E**), and transwell assay (**G**). HCT116 cells were harvested and subcutaneously injected into nude mice for xenografts assay (**I**). Tumor volume (**J**) and the weight (**K**) were calculated, and all date are shown as mean ± SD with five replicates. HCT116 and SW480 cells were transfected indicated constructs. Then, cells were harvested for western blotting and RT-qPCR analysis (**B**), MTS assay (**D**), colony formation assay (**F**) and transwell assay (**H**). Data presented as mean ± SD with three replicates, except for panel **I**–**K**. Student’s *t-*test and one-way ANOVA were used to determine the statistical significance. *p < 0.05; **p < 0.01; ***p < 0.001.

We assessed the role of ZDHHC1 in CRC cell growth by silencing ZDHHC1 expression in HCT116 and SW480 cells using two different shRNAs (Fig. 2A), followed by MTS assay and colony formation analysis. ZDHHC1 silencing significantly promoted cancer cell proliferation (Fig. 2C, E). Additionally, transwell assays revealed that ZDHHC1 knockdown markedly impaired the invasion ability of cancer cells (Fig. 2G). To determine the impact of ZDHHC1 knockdown on colorectal cell growth in vivo, we subcutaneously injected HCT116 cells (expressing shControl or shZDHHC1#2) into nude mice and assessed tumor growth. We found that ZDHHC1 knockdown profoundly induced tumor growth (Fig. 2I–K). Conversely, ectopic overexpression of ZDHHC1 (Fig. 2B) inhibited CRC cell proliferation and invasion, both in HCT116 and in SW480 cells (Fig. 2D, F, H). These findings highlight the key role of ZDHHC1 in CRC progression.

### LIPG is negatively regulated by ZDHHC1 and plays a key role in CRC cell growth through lipid storage

**Fig. 3:**
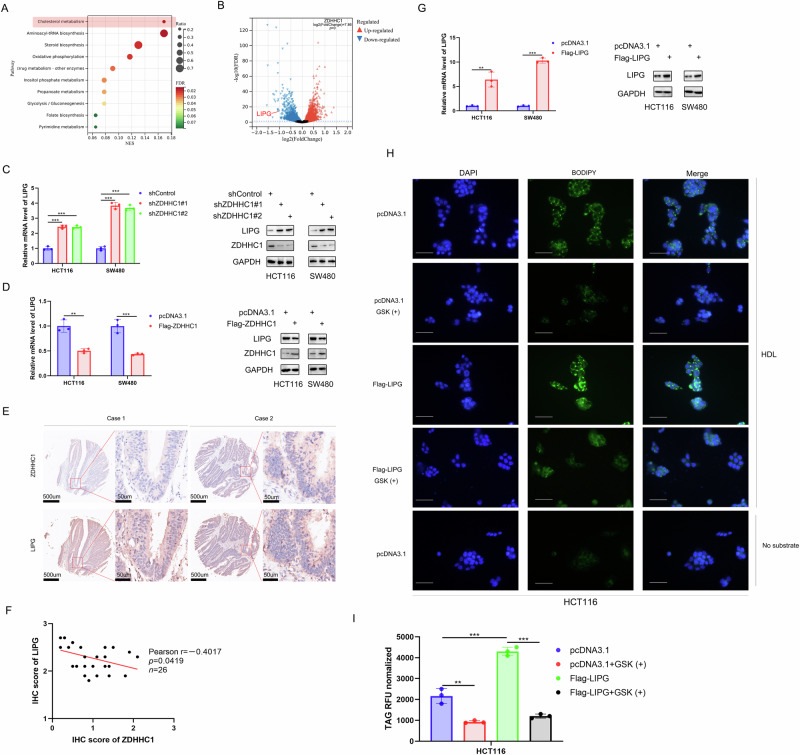
LIPG is negatively regulated by ZDHHC1 and plays a key role in CRC cell growth through lipid storage. HCT116 cells were subjected to RNA-seq, subsequent KEGG pathway enrichment (**A**) and volcano plots analysis (**B**). Cells with knock-down (**C**) and overexpression (**D**) of ZDHHC1were harvested for western blotting and RT-qPCR analysis. The typical IHC images of CRC tissue microarray stained with ZDHHC1 and LIPG (**E**). The size of the scale bar on microscopy images as indicated in the figure. The correlation analysis between ZDHHC1 and LIPG in CRC tissues (**F**). Pearson correlation was used to determine statistical significance; the *P*-value was indicated in the figure. Western blot and RT-PCR were used to analyze LIPG protein and mRNA expression (**G**). HCT116 cells were incubated for 48 h with 800 µg HDL in serum-free DMEM and no substrate with or without GSK (32 nM) (**H**). Lipid droplets were visualized with Bodipy 493/503 staining (green). Nuclei were stained with DAPI (blue). Scale bar, 40 μm. Intracellular triacylglyceride (TAG) levels were quantified with the triglyceride quantification assay kit (**I**). Data presented as mean ± SD with three replicates. Student’s *t-*test and one-way ANOVA were used to determine the statistical significance. *p < 0.05; **p < 0.01; ***p < 0.001.

We conducted RNA sequencing analysis in HCT116 cells with ZDHHC1 overexpression to explore the mechanisms underlying ZDHHC1 antitumor functions (Table S1, S2). KEGG enrichment analysis showed that the cholesterol metabolism pathway was important and this pathway was selected for mapping in response to ZDHHC1 overexpression (Fig. 3A). We also found that there was significant negative association between ZDHHC1 and LIPG (Fig. 3B) and that ZDHHC1 knockdown increased LIPG protein and mRNA expression in HCT116 and SW480 cells (Fig. 3C). Conversely, ZDHHC1 overexpression decreased LIPG levels in CRC cell lines (Fig. 3D). We also evaluated ZDHHC1 and LIPG protein expression in a CRC tissue microarray (*n* = 26 samples) and found an inverse association between the two (Pearson correlation *r* = −0.4017, *p* = 0.0419; Fig. 3E, F).

Next, we assessed the function of LIPG in CRC cell growth. We silenced LIPG expression in HCT116 and SW480 cells using two different shRNAs (Fig. S2A, B), followed by MTS assay and colony formation analysis. LIPG silencing significantly inhibited cancer cell proliferation (Fig. S2C, E). Additionally, transwell assays revealed that LIPG knockdown markedly impaired the invasion ability of cancer cells (Fig. S2G). Conversely, ectopic overexpression of LIPG (Fig. 3G) enhanced CRC cell proliferation and invasion, both in HCT116 and in SW480 cells (Fig. S2D, F, H). Thus, we propose that LIPG is negatively regulated by ZDHHC1 and plays a key role in CRC cell growth.

Previous work has shown that LIPG exerts phospholipase A1 activity towards high density lipoprotein (HDL)-derived phosphatidylcholine (PC) [19], releasing lipid products that become incorporated into intracellular PC and triacylglyceride (TAG) pools [20]. Therefore, we investigated whether this also applies to CRC cells. LIPG overexpression in the presence of HDL resulted in an increase in intracellular TAG levels, as well as lipid droplet (LD) accumulation compared to cells transfected with vector alone (Fig. 3H, I). Blockage of LIPG activity with the LIPG inhibitor GSK264220A [21] significantly reduced intracellular TAG levels and LD accumulation in HCT116 cells. LIPG in the absence of substrate did not elicit such a response, suggesting that both LIPG and substrate are required to increase the intracellular TAG pool.

### ZDHHC1 interacts with LIPG in CRC cells

**Fig. 4:**
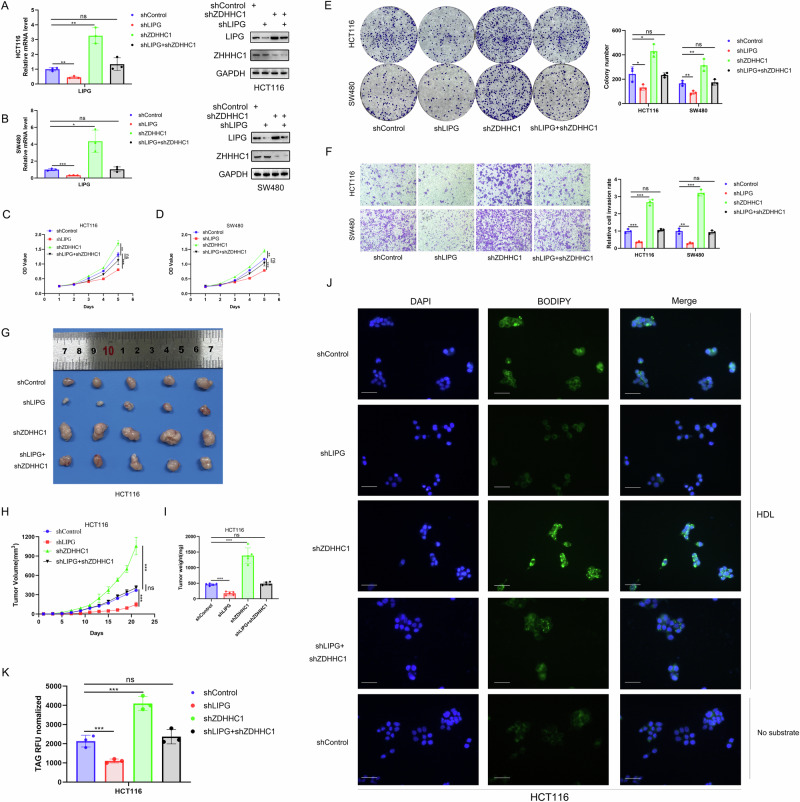
ZDHHC1 interacts with LIPG in CRC cells. Cells with shControl, shZDHHC1, shLIPG, and shZDHHC1/shLIPG were harvested for western blotting and RT-qPCR analysis (**A**, **B**). Proliferation, colony formation assay and transwell invasion with HCT116 and SW480 cells were performed in each group (**C**–**F**). HCT116 cells were harvested and injected into nude mice for xenografts assay (**G**). Tumor volume (**H**) and the weight (**I**) were calculated, and all date are shown as mean ± SD with five replicates. LD were visualized with Bodipy 493/503 staining (green) (**J**). Scale bar, 40 μm. Intracellular TAG levels were quantified with the triglyceride quantification assay kit (**K**). Data presented as mean ± SD with three replicates. Student’s *t-*test and one-way ANOVA were used to determine the statistical significance. *p < 0.05; **p < 0.01; ***p < 0.001.

To assess the importance of LIPG for the anti-tumor effects of ZDHHC1 in CRC, we used HCT116 and SW480 cells expressing shControl, shZDHHC1, shLIPG, and shZDHHC1/shLIPG (Fig. 4A, B). ZDHHC1 silencing increased tumor growth, and LIPG silencing alone inhibited the proliferation and invasion of CRC cells (Fig. 4C–F). We also conducted in vivo experiments to analyse the effect of ZDHHC1 and LIPG on tumorigenicity. Transfected cells were injected into the flanks of nude mice to form ectopic tumors. After 21 days, we observed impaired tumor growth in the shLIPG group and induced tumor growth in the shZDHHC1 group (Fig. 4G, H). Average tumor weight was calculated (Fig. 4I). Knock-down of ZDHHC1 resulted in an increase in intracellular TAG levels and LD accumulation, while LIPG silencing alone showed opposite results (Fig. 4J, K). Notably, combined silencing of ZDHHC1 and LIPG diminished the proliferative effects of ZDHHC1 downregulation in vitro and in vivo (Fig. 4A–K), indicating that ZDHHC1 inhibits CRC progression by targeting LIPG for down-regulation.

### IGF2BP1 regulates LIPG mRNA stability via m6A modification

**Fig. 5:**
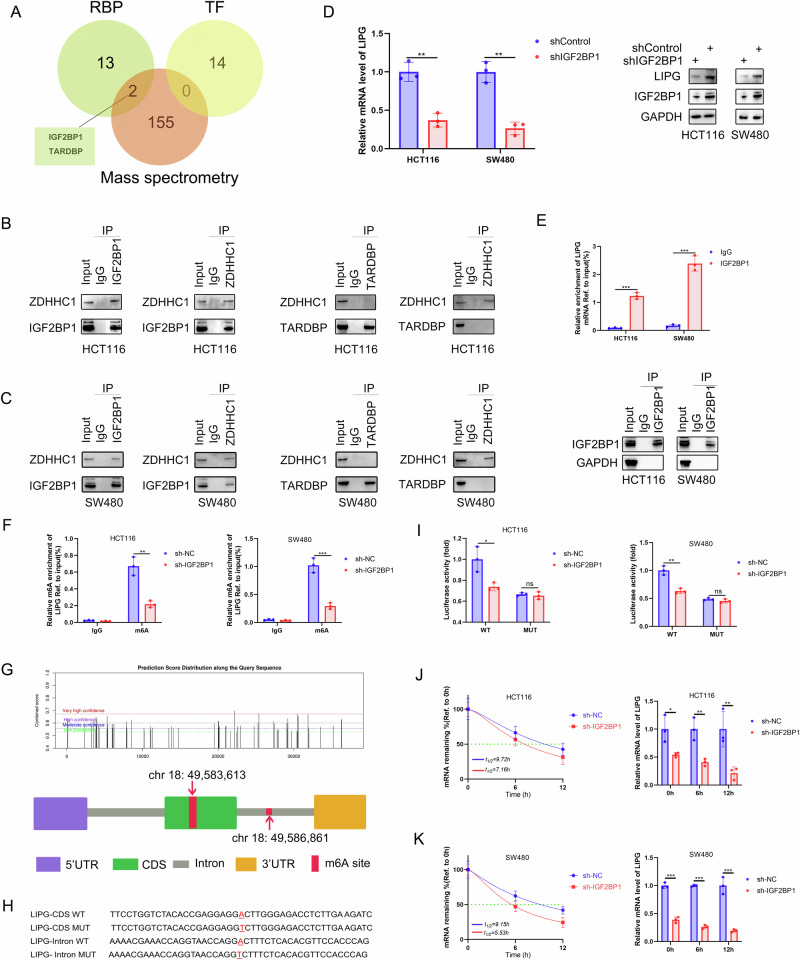
IGF2BP1 regulates LIPG mRNA stability via m6A modification. Venn diagram showed IGF2BP1 and TARDBP were the potential binding partners of ZDHHC1 (**A**). Co immunoprecipitation analysed and verified the interaction between ZDHHC1 and IGF2BP1 in HCT116 and SW480 cells (**B**, **C**). IGF2BP1 knockdown decreased the protein and mRNA level of LIPG by western blotting and RT-qPCR analysis (**D**). RIP-qPCR analysis showing the enrichment of LIPG mRNA in anti-IGF2BP1 precipitates (**E**). MeRIP-qPCR analysis showing the m6A enrichment of LIPG mRNA in HCT116 and SW480 cells (**F**). Two very high-confidence m6A site was identified upon LIPG mRNA based on the SRAMP software analysis (**G**). Schematic representation of LIPG- WT (wild-type) or LIPG- MT (mutated-type) sequence (**H**). Luciferase reporter assays measured the luciferase activities of LIPG WT or LIPG Mut in CRC cells with IGF2BP1 knockdown (**I**). After silencing IGF2BP2 in HCT116 and SW480 cells, the mRNA half-lives and expression of LIPG were analyzed at the predetermined times following actinomycin D (5 μg/ml) treatment (**J**, **K**). Data presented as mean ± SD with three replicates. Student’s *t*-test was used to determine the statistical significance. *p < 0.05; **p < 0.01; ***p < 0.001.

To elucidate the mechanisms underlying the regulatory activity of ZDHHC1 on LIPG in CRC, we performed immunoprecipitation and mass spectrometry to identify binding partners of ZDHHC1 (listed in Table S3). We investigated the top 20 transcription factors (TFs) (https://chip-atlas.org) and RNA-binding proteins (RBPs) (https://rnasysu.com/encori/) related to LIPG genes and intersected them with the mass spectrometry results. Two RBPs (IGF2BP1 and TARDBP) were identified as potential binding partners of ZDHHC1 (Fig. 5A). Co-immunoprecipitation results confirmed the interaction between ZDHHC1 and IGF2BP1 in HCT116 and SW480 cells (Fig. 5B, C). Since IGF2BP1 is considered to be an RBP, also known as “reader” in m6A RNA modification [22, 23], we investigated whether IGF2BP1 interacts with LIPG via m6A modification. First, we found that IGF2BP1 knockdown decreased the protein and mRNA level of LIPG (Fig. 5D). Next, we performed RIP-qPCR assays using the anti-IGF2BP1 antibody in HCT116 and SW480 cells, and the results showed significant enrichment of LIPG mRNA compared to the IgG control group (Fig. 5E). To verify whether LIPG is affected by m6A modification, we conducted MeRIP-qPCR assays and found that knockdown of IGF2BP1 markedly decreased LIPG m6A levels in CRC cells compared with corresponding control cells (Fig. 5F). Based on SRAMP software analysis, we identified two high-confidence m6A site in the Coding Sequence (CDS) and introl region of LIPG mRNA (Fig. 5G). To validate these findings, we performed luciferase reporter assays using a luciferase reporter containing a wildtype (WT) LIPG or mutated-type (MT) LIPG sequence (Fig. 4H). As expected, luciferase activity was significantly attenuated in the LIPG WT assay when IGF2BP1 was silenced, while LIPG-MT seemed to be unaffected (Fig. 5I). Furthermore, mRNA stability assays revealed that the mRNA expression of LIPG was decreased and the mRNA half-life of LIPG was continually reduced by IGF2BP1 silencing in both HCT116 and SW480 cells (Fig. 5J, K). Overall, these findings suggest that IGF2BP1 directly binds to and stabilizes LIPG mRNA in an m6A modification-dependent manner.

### ZDHHC1 is a IGF2BP1-palmitoylating enzyme that induces IGF2BP1-C337 S-palmitoylation

**Fig. 6:**
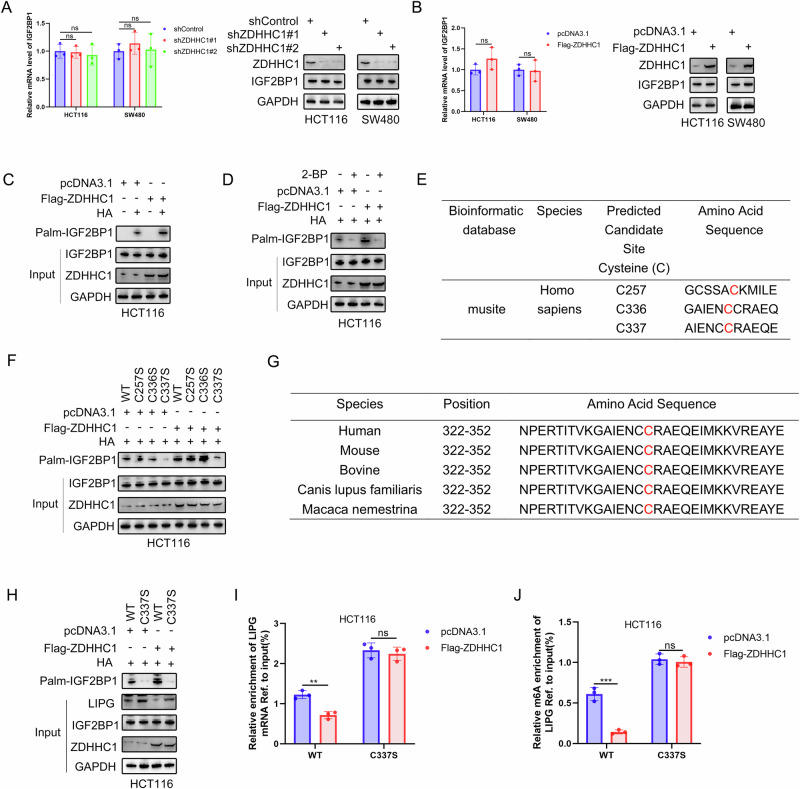
ZDHHC1 is a IGF2BP1-palmitoylating enzyme that induces IGF2BP1-C337 S-palmitoylation. ZDHHC1 could not regulate the protein and mRNA level of IGF2BP1 by western blotting and RT-qPCR analysis (**A**, **B**). IP-ABE analysis of IGF2BP1 palmitoylation in HCT116 cells when treated with or without palmitoylation inhibitor 2-BP (**C**, **D**). Predicted cysteine residues on IGF2BP1 susceptible to S-palmitoylation (**E**). IP-ABE analysis of IGF2BP1 palmitoylation in HCT116 cells when ectopically expressing IGF2BP1-WT or harboring specific mutations (**F**). Alignment of the similarity of PCSK9 sequences in different vertebrate orthologs (**G**). Flga-ZDHHC1 reduce the level of LIPG with IGF2BP1-WT but not IGF2BP1-C337S by western blotting analysis (**H**). RIP-qPCR analysis showing the enrichment of LIPG mRNA in anti-IGF2BP1 precipitates (**I**). MeRIP-qPCR analysis showing the m6A enrichment of LIPG mRNA in HCT116 cells (**J**). Data presented as mean ± SD with three replicates. Student’s *t-*test and one-way ANOVA were used to determine the statistical significance. *p < 0.05; **p < 0.01; ***p < 0.001.

Our data showed that ZDHHC1 did not regulate the protein and mRNA levels of IGF2BP1 (Fig. 6A, B). Previous data suggest that ZDHHC1 is a palmitoyl acyltransferase regulating the palmitoylation of various proteins [24]; therefore, we speculated that a IFG2BP1 cysteine may be palmitoylated by ZDHHC1. Therefore, cells of HCT116-expressing FLAG-ZDHHC1 were used to assess protein palmitoylation status via the acyl-biotin exchange (ABE) technique, which revealed that IGF2BP1 was palmitoylated in CRC cells (Fig. 6C). The increase in palmitoylated IGF2BP1 was almost completely negated in the presence of palmitoylation inhibitor 2-bromopalmitate (2-BP) (Fig. 6D). To identify the cysteine residue(s) in IGF2BP1 that had been palmitoylated by ZDHHC1, we utilized the palmitoylation site prediction tool (https://www.musite.net/). Among the potential cysteine residues within IGF2BP1, C257, C336 and C337 had the highest scores (Fig. 6E). Based on this, we constructed various IGF2BP1 mutants; however, only the C337S mutation abolished palmitoylation of IGF2BP1 (Fig. 6F). Moreover, the C337 palmitoylation site was found to be highly conserved in different animal species (Fig. 6G). We also found that Flga-ZDHHC1 reduced the level of LIPG in IGF2BP1 wild type (WT) but not IGF2BP1-C337S (Fig. 6H–J). Taken together, these data suggest that ZDHHC1 may be an IGF2BP1-palmitoylating enzyme that induces S-palmitoylation at IGF2BP1-C337 to downregulate the expression of LIPG.

### The ZDHHC1/IGF2BP1/LIPG signaling axis inhibits CRC cell growth

**Fig. 7:**
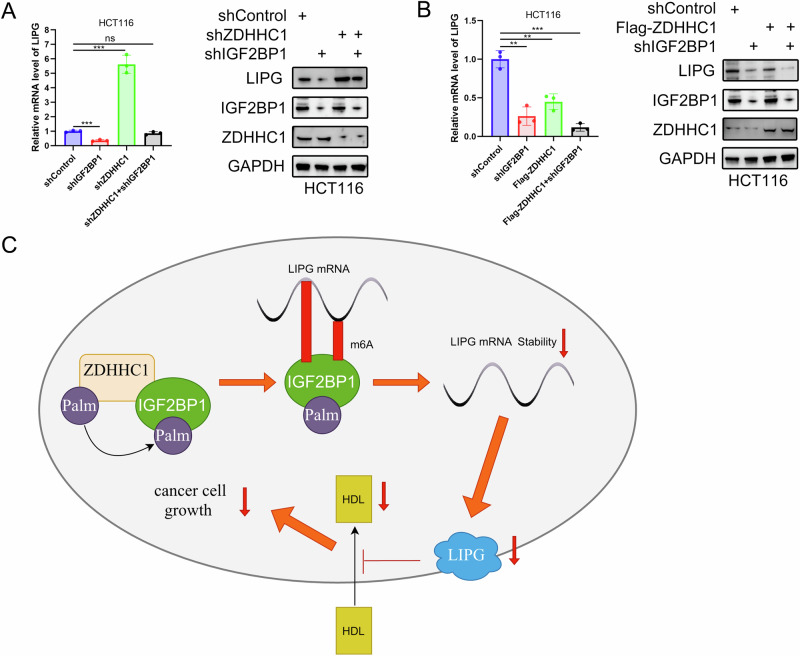
The ZDHHC1/IGF2BP1/LIPG signaling axis inhibits CRC cell growth. HCT116 cells were infected with indicated shRNAs and harvested for western blotting analysis and RT-qPCR analysis (**A**). HCT116 cells were infected with indicated shRNAs and plasmids, then harvested for western blotting analysis and RT-qPCR analysis (**B**). A graphic illustration of the proposed mechanism in this study (**C**). In brief, IGF2BP1 is palmitoylated by ZDHHC1, and then bound to the m6A site upon LIPG mRNA to reduce the stability and expression of LIPG mRNA, thereby inhibiting CRC cells from taking HDL, and leading to the decrease of CRC cell growth. Data presented as mean ± SD with three replicates. One-way ANOVA was used to determine the statistical significance. *p < 0.05; **p < 0.01; ***p < 0.001.

Next, we silenced ZHDDC1, IGF2BP1, and ZHDDC1/ IGF2BP1 expression in HCT116 cells. ZDHHC1 knockdown upregulated LIPG expression levels; however, double knockdown of ZDHHC1 and IGF2BP1 abolished the ability of ZDHHC1 to regulate LIPG expression (Fig. 7A). Moreover, ZDHHC1 overexpression decreased LIPG expression in HCT116 cells, both at the protein and mRNA levels (Fig. 7B). We also found that IGF2BP1 knockdown increased ZDHHC1-mediated LIPG downregulation (Fig. 7B). Overall, these data indicate that ZDHHC1 downregulates LIPG expression and inhibits CRC growth by palmitoylation of IGF2BP1 (Fig. 7C).

## Discussion

Here, we show that ZDHHC1 may be a central component of metabolism in CRC cells by specifically downregulating LIPG expression, thereby downregulating the acquisition of indispensable intracellular lipid HDL for CRC proliferation. The ZDHHC family of proteins are abundant in human cells and may act as either oncoproteins or tumor suppressors. In CRC, ZDHHC1 acts as a tumor suppressor when at high levels. Moreover, we show that ZDHHC1 inhibits CRC growth through palmitoylating IGF2BP1, which results in reduced stability of LIPG mRNA in an m6A dependent-manner.

In the past decade, palmitoylation has been shown to be implicated in various aspects of cancer, including cancer cell proliferation, invasion, metastasis, and antitumor immunity [15, 25]. Previous studies have reported that 26% of cancer driver gene encoded proteins can be palmitoylated [15, 26]. One of the prominent distinctions between tumor cells and normal cells is that tumor cells exhibit an expanded metabolic reservoir that is sufficiently flexible to withstand and grow in the harsh tumor micro-environment [27]. The importance of adipose tissue in cancer metabolism is reinforced by the finding that obesity plays a catalytic role in tumorigenesis in breast, ovarian, and pancreatic cancers [28]. Obesity in CRC appears to increase the risk of disease progression and mortality. Tumors tend to develop around fat‐rich tissues with adipocytes and cancer cells establishing a symbiotic relationship [29], and it is possible that individual ZDHHC enzymes in humans could act as either oncoproteins or tumor suppressors in a tissue-specific manner [15]. Our study shows that ZDHHC1, acting as a tumor suppressor gene, inhibits the growth of CRC cells and downregulates the expression of LIPG through palmitoylation of IGF2BP1. These findings suggest that ZDHHC1 might be valuable for the prognosis of CRC. Metabolic reprogramming plays an essential role in the proliferation and survival of cancer cells [30]. Our findings further confirm the significance of lipid metabolism during the process of ZDHHC1 regulation in CRC.

LIPG is an important hydrolase that helps to regulate HDL in the blood, and exerts its function via binding to proteoglycan on the cell surface [31]. Studies have demonstrated that LIPG plays an important role in the initiation and development of various malignant tumors such as breast cancer, gastric cancer, and testicular cancer [32–35]. Compared to normal cells, cancer cells undergo unlimited proliferation. Chen et al. revealed that LIPG supports cell proliferation and growth in malignant tumors such as breast cancer by utilizing lipids for energy provision [36]. However, there are no reports to date on the potential role of LIPG in CRC. Our study showed that LIPG can promote CRC proliferation by increasing intracellular lipid storage, which suggests that mechanisms of lipid metabolism may be conserved regardless of tumor location. In addition, decreased HDL in the blood may be a risk factor for CRC. Orlistat is a Food and Drug Administration (FDA)-approved anti-obesity drug that inhibits fatty acid synthase (FASN). Data have shown that Orlistat may block epidermal growth factor receptor (EGFR) palmitoylation, alter EGFR cellular distribution, induce EGFR ubiquitination, and reduce tumorigenesis in vivo and in vitro [37]. Our study demonstrates that the LIPG inhibitor, GSK264220A, is able to reverse the activity of LIPG on HDL; therefore, we propose that anti-lipid metabolism drugs may represent a promising therapeutic strategy for the treatment for CRC in the future. Since high expression of LIPG is detected in CRC, it is possible to apply LIPG for a potential and auxiliary tumor biomarker and cancer gene therapy. However, more studies are needed before its amplification clinically.

Our study has several limitations. Firstly, the number of specimens was small as CRC tumor tissue is difficult to access. Secondly, although our findings reveal that ZDHHC1 downregulates LIPG by IGF2BP1 palmitoylation, we did not explore how palmitoylation of IGF2BP1 affects LIPG m6A levels. Thus, a clearer understanding of detailed mechanisms must be sought in future studies.

In summary, our study reveals the clinical and biological function of ZDHHC1 in CRC cells for the first time. We demonstrated that ZDHHC1 inhibits CRC growth by reducing the stability of LIPG mRNA in an m6A dependent-manner by palmitoylation of IGF2BP1, which indicates that the ZDHHC1/IGF2BP1/ LIPG signaling axis may be an important mediator of CRC progression, and inhibiting LIPG may be a potential strategy to prolong the survival of patients with CRC.

## Supplementary information

Supplementary figure

Table S1

Table S2

Table S3

## Data Availability

All data are available in the main text or the supplementary materials.
