# Supplementary material for: ZDHHC1 downregulates LIPG and inhibits colorectal cancer growth via IGF2BP1 Palmitoylation
Source: Cancer Gene Ther. 2024 Jul 28;31(9):1427–37. doi: 10.1038/s41417-024-00808-1 (PMC11405259; doi:10.1038/s41417-024-00808-1)
Supplement: Supplementary file 1 — Supplementary figure [file 41417_2024_808_MOESM1_ESM.docx]

**Supplemental Figure**

ZDHHC1 downregulates LIPG and inhibits colorectal cancer growth via IGF2BP1 Palmitoylation

Qun Zhang^1#^, Zhouyuan Du^1#^, Wei Zhou^1^, Wei Li^1^, Qinglin Yang^1^, Haixin Yu^1*^, Tao Liu^1*^

**Affiliations**

^1^Department of Digestive Surgical Oncology, Union Hospital, Tongji Medical College, Huazhong University of Science and Technology, Wuhan 430022, China





Fig. S1. Three-years overall CRC survival. Twenty members of ZDHHC family with three-years overall CRC survival were analyzed via TIMER 2.0.


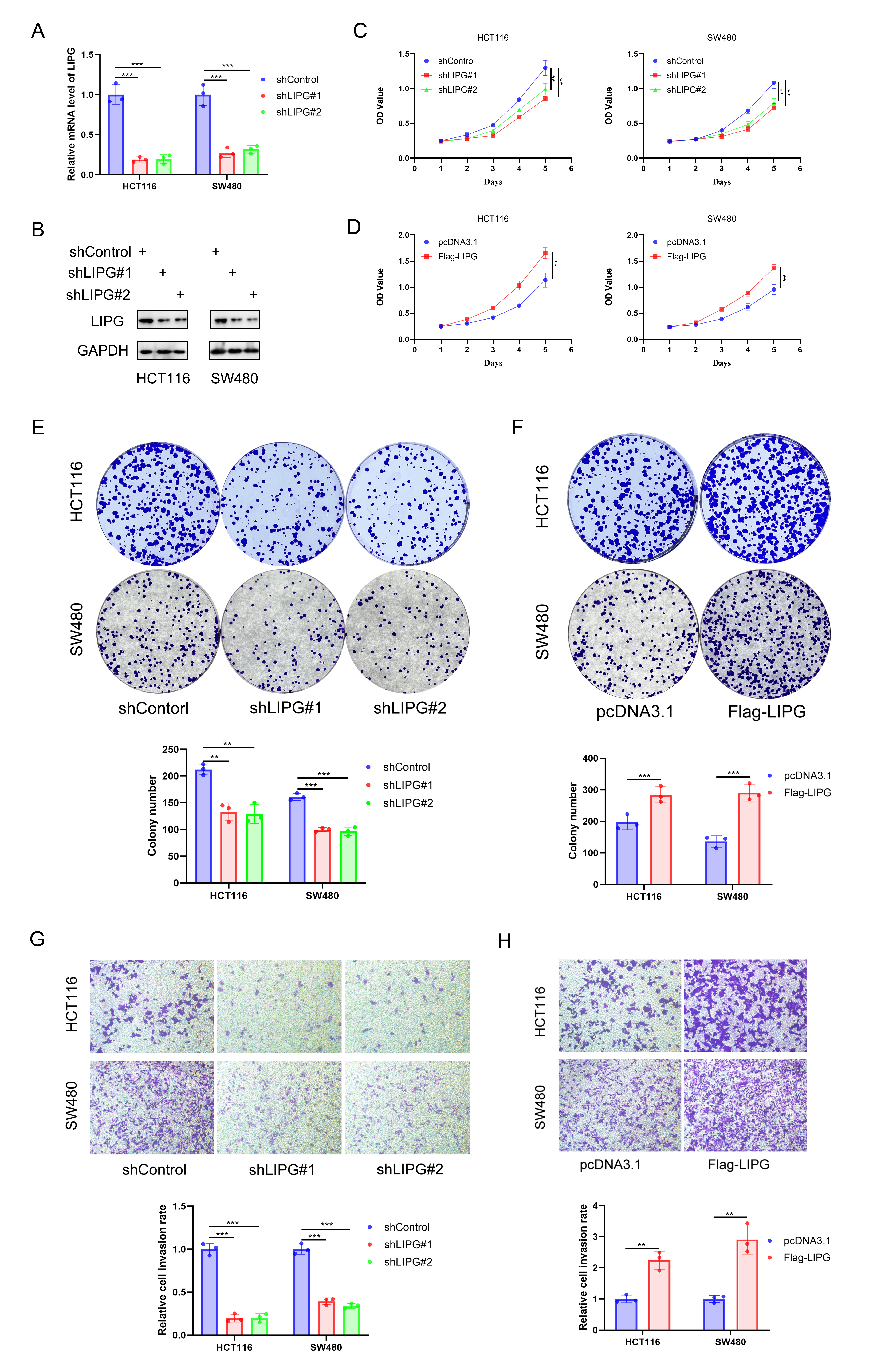


Fig. S2. LIPG promotes CRC cell proliferation and migration. HCT116 and SW480 cells were infected with indicated shRNAs. Cells were harvested for western blotting and RT-qPCR analysis (A, B), MTS assay (C), colony formation assay (E), and transwell assay (G). HCT116 and SW480 cells were transfected with a LIPG constructs. Then, cells were harvested for MTS assay (D), colony formation assay (F) and transwell assay (H). Data presented as mean±SD with three replicates. Student’s t test and one-way ANOVA were used to determine the statistical significance. *, p< 0.05; **, p < 0.01; ***, p < 0.001.
